# Dynamic Regulation of Hepatic Lipid Droplet Properties by Diet

**DOI:** 10.1371/journal.pone.0067631

**Published:** 2013-07-11

**Authors:** Amanda E. Crunk, Jenifer Monks, Aya Murakami, Matthew Jackman, Paul S. MacLean, Mark Ladinsky, Elise S. Bales, Shannon Cain, David J. Orlicky, James L. McManaman

**Affiliations:** 1 Graduate Program of Molecular Biology, University of Colorado School of Medicine, Aurora, Colorado, United States of America; 2 Division of Basic Reproductive Sciences, University of Colorado School of Medicine, Aurora, Colorado, United States of America; 3 Division of Endocrinology and Metabolism, University of Colorado School of Medicine, Aurora, Colorado, United States of America; 4 The Center for Human Nutrition, University of Colorado School of Medicine, Aurora, Colorado, United States of America; 5 The Colorado Obesity Research Initiative, University of Colorado School of Medicine, Aurora, Colorado, United States of America; 6 The Boulder Laboratory for 3D Electron Microscopy, University of Colorado Boulder, Boulder Colorado, United States of America; 7 Department of Pathology, University of Colorado School of Medicine, Aurora, Colorado, United States of America; Nebraska Medical Center, United States of America

## Abstract

Cytoplasmic lipid droplets (CLD) are organelle-like structures that function in neutral lipid storage, transport and metabolism through the actions of specific surface-associated proteins. Although diet and metabolism influence hepatic CLD levels, how they affect CLD protein composition is largely unknown. We used non-biased, shotgun, proteomics in combination with metabolic analysis, quantitative immunoblotting, electron microscopy and confocal imaging to define the effects of low- and high-fat diets on CLD properties in fasted-refed mice. We found that the hepatic CLD proteome is distinct from that of CLD from other mammalian tissues, containing enzymes from multiple metabolic pathways. The hepatic CLD proteome is also differentially affected by dietary fat content and hepatic metabolic status. High fat feeding markedly increased the CLD surface density of perilipin-2, a critical regulator of hepatic neutral lipid storage, whereas it reduced CLD levels of betaine-homocysteine S-methyltransferase, an enzyme regulator of homocysteine levels linked to fatty liver disease and hepatocellular carcinoma. Collectively our data demonstrate that the hepatic CLD proteome is enriched in metabolic enzymes, and that it is qualitatively and quantitatively regulated by diet and metabolism. These findings implicate CLD in the regulation of hepatic metabolic processes, and suggest that their properties undergo reorganization in response to hepatic metabolic demands.

## Introduction

Obesity, which is largely driven by excess calorie consumption, affects nearly 30% of the population in the United States [1] and is a chief etiological factor in development of metabolic disorders that underlie type II diabetes, cardiovascular and kidney diseases and non-alcoholic fatty liver disease [2]. As a major regulator of energy homeostasis, the liver is a primary target of obesity-associated metabolic alterations [3]; and disruption of hepatic lipid metabolism is proposed to play a fundamental role in the initiation and progression of many metabolic diseases [4], [5].

Abnormal intrahepatic fat accumulation (steatosis) in the form of cytoplasmic lipid droplets (CLD) is an early pathophysiological feature of altered liver metabolism that is linked to insulin resistance and potential progression to severe liver disease [6], [7]. CLD are organelle-like structures that are critically involved in the storage, trafficking and metabolic regulation of cellular neutral lipids [8]. Consequently, understanding how CLD affect hepatic metabolism, and how nutritional status impacts their functions, are important elements in defining the mechanistic links between hepatic steatosis and metabolic diseases. CLD are composed of a neutral lipid core, surrounded by a phospholipid monolayer, and coated by various proteins that regulate CLD functional properties [9]. Members of the perilipin (PLIN) family are among the most abundant CLD-associated proteins in mammalian cells, functioning as physiologically important regulators of cellular lipid metabolism and trafficking [10]. Gene disruption studies in mice have documented that perilipin-2 (Plin2, adipophilin, ADRP) is required for hepatic lipid accumulation in response to high fat diet (HFD) feeding [11]–[13]. However, other PLIN family members have been detected on hepatic CLD in humans and mice with fatty liver disease [14], [15], which suggests the possibility that diet and/or altered metabolic properties can dynamically influence hepatic CLD protein composition.

Protein compositions of CLD been characterized to varying degrees from multiple sources including; yeast (*Saccharomyces cerevisiae*) [16], [17], *Drosophila* [18]–[20], mouse mammary epithelial cells [21], Chinese hamster ovary K2 cell lines [22], [23], 3T3-L1 adipocytes [24], [25], cultured human A431 epithelial cells [26], HuH7 human hepatoma cell line [27], cultured human hepatocyte HepG2 cell lines [28], liver tissue from Sprague-Dawley rats [29] and mice [21], human lymphoblast U937 cells from lung tissue [30], and mouse skeletal muscle [31]. However, information about the protein composition of hepatic CLD remains relatively limited, and the effects of diet and/or metabolic alterations on CLD protein properties are not known in detail for any tissue.

The highly dynamic and adaptive nature of liver metabolism is sensitive to nutrient status [32]. In rodents, fasting and refeeding is associated with alterations in hepatic glucose and lipid metabolism that are reflected in increased expression of lipid metabolizing genes and the accumulation of intrahepatic lipids [33], [34]. The nature of these responses is influenced by the amount of and types of dietary fat [35]. In the work presented here, we use a fasting and refeeding model to test the hypothesis that the hepatic CLD proteome is influenced by dietary fat composition. Our data show that low fat (LF) and high fat (HF) diets differentially affect the types and quantities of CLD associated protein compositions, and that these differences are associated with differences in hepatic metabolism and CLD properties. Taken together these findings indicate the hepatic CLD proteome is dynamically regulated by the nutrient and metabolic status of the liver and provide evidence that CLD may function as a platform for regulating hepatic metabolic activity.

## Methods

### Ethics Statement

All procedures involving animals were performed in accordance with published National Institutes of Health Guidelines. The University of Colorado Anschutz Medical Campus Institutional Animal Care and Use Committee approved this study and all procedures and housing conditions used to complete it.

### Materials

Chemicals used were purchased from Sigma Chemical Company (St. Louis, MO). Antibodies to N- and C-terminal regions of Plin2 and to Plin3 were raised in rabbits as described [36]. Guinea pig antibodies specific to the N-terminal 25 amino acids of mouse Plin2 were purchased from Fitzgerald (North Acton, MA). Rabbit antibodies to GRP78 and PDI were purchased from Novus Biologicals (Littleton, Colorado), and Fitzgerald Inc. (North Acton, MA) respectively. Rabbit anti-BHMT was purchased from Santa Cruz Biotechnology (Santa Cruz, CA). Horseradish-peroxidase-conjugated and Alexfluor-conjugated secondary antibodies were purchased from Life Technologies (Grand Island, NY). IR dye-conjugated secondary antibodies were purchased from Li-COR Biosciences (Lincoln, Nebraska). High fat (60 kcal%; D12492) and low fat (10 kcal%, D12045B) diets were purchased from Research Diets Inc. (New Brunswick, NJ).

### Animal procedures

Twelve-week-old C57BL/6 mice were fasted for 24 hours, with access to water *ad libitum*, and refed with LF (10.0% fat-derived calories, 24.0% protein-derived calories and 60% carbohydrate-derived calories) or HF (60.3% fat-derived calories, 18.4% protein-derived calories and 21.3% carbohydrate-derived calories) diets for 16 hours. Livers were removed from refed animals, euthanized by carbon dioxide, and weighed. A portion of each liver was removed and used for CLD isolation. The remaining portions were fixed in 4% paraformaldehyde and processed for paraffin imbedding [15] or flash frozen in liquid nitrogen for RNA and protein analyses.

### Metabolic monitoring

Three separate cohorts of mice were placed in a metabolic monitoring system that provided measurements of energy balance (intake and expenditure), the respiratory exchange ratio (RER), and activity levels (Columbus 8M Oxymax) [37]. Mice were individually housed in metabolic chambers and allowed to acclimate for one week prior to fasting and refeeding.

### CLD isolation

CLD were isolated from liver homogenates by sucrose gradient centrifugation as described previously [21]. Briefly, freshly dissected livers were homogenized on ice in an equal volume of ice cold homogenization buffer (37.5 mM TRIS-malate, pH 6.4; 0.5 M sucrose; and 5 mM MgCl_2_ pH 6.4 plus protease inhibitors (Aprotinin, Leupeptin, Peptstatin, AEBSF, PIC1, PIC2 ) using a Dounce homogenizer. The homogenate was centrifuged at 3000× g for 10 minutes at 4°C. CLD were isolated from the resulting postnuclear supernatant fraction by sucrose gradient centrifugation and washed by repeated (3X) floatation as described [21]. For proteomics analysis the CLD sample was diluted in 100 ul of 10 mM Tris pH 7.4. For western blot analysis the samples were diluted in 100 ul of 5% SDS in 10 mM Tris pH 7.4 plus protease inhibitors and stored in −80°C.

### RNA extraction and transcript quantitation

Total RNA was extracted from frozen tissue using Trizol (Life Technologies) according to the manufacturer's instructions. The purity, concentration, and integrity of total RNA from each sample were verified using a NanoDrop spectrophotometer (NanoDrop Technologies, Wilmington, DE). Transcript copy numbers were determined by quantitative real-time (QRT)-PCR analysis using a multiplexing strategy to provide an internal standard for normalization (18s ribosomal). QRT-PCR assays were performed in the Quantitative Genomics Core Laboratory at the University of Texas Health Sciences Center as described previously [36] using validated primers and probes shown in Table S1. At least three tissue replicates were analyzed at least twice with similar results.

### Protein extraction and quantitation

Protein concentrations in extracts and isolated fractions were measured using Bio-Rad Protein Assay (Hercules, CA). CLD-associated proteins were extracted in 5% SDS and stored at −80°C prior to analysis by SDS-PAGE and immunoblotting. Proteins were separated on 10% polyacrylamide gels and stained with Coomassie blue or transferred to nitrocellulose membranes for immunoblot analysis, using the following primary antibodies and dilutions. Guinea pig anti-Plin2 (1:1,000); rabbit anti-PDI (1:500); rabbit anti-GRP78 (1:1000); rabbit anti-BHMT (1:500). Infrared dye-conjugated secondary antibodies (Li-COR, Lincoln, Nebraska ) were used according to the manufacturer's specifications. Antibody staining intensity was quantified using an Odyssey CLX system.

### In solution digest and LC-MS/MS

Protein from the isolated CLD was precipitated using methanol chloroform (1:2). After the protein pellet was dissolved in 2 ul of 1% Protease Max Surfactant in 50 mM ammonia bicarbonate (ABC) (Promega, Madison, WI), 83.5 ul of ABC was added to the sample followed by DTT to a final concentration of 5 mM. The sample was incubate for 20 minutes at 56°C and then cooled to RT. Iodoacetamide was added to a final concentration of 15 mM and incubated at RT in the dark for 15 minutes to block the sulfhydryl groups. A second addition of 1ul of 1% Protease Max Surfactant was added to the sample followed by 1ug of Trypsin (Promega, Madison, WI). The sample was digested overnight at 37°C. The condensate was collected by centrifugation at 12,000× g for 10 seconds. The reaction was quenched by the addition of trifluroacetic acid (TFA) to a final concentration of 0.5% and incubated for 5 minutes at RT. The samples were purified and concentrated using a 10 ul μ-c18 ZipTip (Millipore, CA) according to the manufactures directions. The peptides were eluted in 60%acetonitrile/0.1% formic acid. HPLC-MS/MS was performed by the University of Colorado, Anschutz Medical Campus Mass Spectrometry/Proteomics Core Facility. Samples were analyzed by microcapillary HPLC tandem mass spectrometry (μLC-MS/ΜS) using an LTQ XL mass spectrometer (Thermo, San Jose, CA). Samples (2.5 μL) were injected onto a reverse-phase column via a cooled (12°C) autosampler (Eksigent, Dublin, CA) connected to an HPLC system (Agilent 1100, Agilent Technologies, Santa Clara CA) that was set at 70 μL/min before the split and ∼350 nL/min after the split. HPLC buffers used were Buffer A: 94.9% water, 5% acetonitrile, and 0.1% formic acid and Buffer B: 94.9% acetonitrile, 5% water, and 0.1% formic acid. A 90-minute HPLC gradient was used to separate peptides. The gradient changed from 5% to 28% acetonitrile over 60 minutes followed by organic and aqueous washes on a 15 cm microcapillary HPLC column with a pulled 5 μm nanospray tip for nano-electrospray ionization. The column was packed in-house with reverse-phase stationary phase Synergi 4u, 100 Å C_18_ (Phenomenex, Torrance, CA). The column was heated to 60°C using a column heater constructed in-house.

### Data acquisition

Mass spectrometry data acquisition was performed in data-dependent mode on the Xcalibur instrument software (v. 2.0.6, Thermo, San Jose, CA) with a single MS1 scan (30 ms) followed by up to three data dependent collision induced dissociation scans (MS/MS, 30 ms each). Data were converted from the Thermo *.raw data file format to the *.mgf format using an in-house script. After conversion, data were searched against the mouse Swissprot database (downloaded 12/14/2011) using Mascot® (v. 2.2.07, Matrix Science Ltd., Boston, MA). For searches, mass tolerances were set at ±0.60 Da for both MS peaks and MS/MS fragment ions. Trypsin enzyme specificity was applied allowing one missed cleavage in the database searches. Modifications searched included fixed carbamidomethyl modification of cysteine and the variable oxidation modifications of methionine, protein N-terminal acetylation, peptide N-terminal pyro-glutamic acid formation. Results from the Mascot searches were analyzed and sorted using Scaffold® (v. 3.00, Proteome Software, Portland, OR).

### Immunohistochemistry and Fluorescence Imaging

Paraffin sections were processed for immunohistochemistry as described previously [38]. Immunoreactivity was visualized using secondary antibodies conjugated with Alexafluor 488 or Alexafluor 594 at dilutions of 1:500 and 1:250 respectively. Nuclei were stained with DAPI (Sigma Chemical Company, St Louis, MO). Immunofluorescence images were captured on a Nikon Diaphot fluorescence microscope.

For CARS microscopy and BODIPY staining, livers from refed animals were perfused with paraformaldehyde, sectioned at 10 um, collected onto Cell-Tak coated coverslips, and vapor-fixed with paraformaldehyde for 20 min before being gently rehydrated with PBS. Auto-fluorescence was quenched with 2 mg/ml glycine for 10 min. Sections were rinsed with PBS and stained with BODIPY 493/503 at a final concentration of 30 ug/ml, and DAPI at 5 ug/ml. Coverslips were mounted in PBS and imaged within 3 days. Confocal imaging of BODIPY 493/503 and DAPI was performed on a 3i Marianas Inverted Spinning Disk Confocal system. All image analyses and rendering were performed using SlideBook Software (Intelligent Imaging Innovations, Inc., Denver, CO). Coherent anti-Stokes Raman scattering (CARS) images of lipid droplets in tissue sections were acquired with a custom-built multiphoton microscopy platform optimized for CARS as previously described [39]. All images were processed by Photoshop (Adobe Systems Inc., Mountain View, CA).

### Immunoelectron microscopy

Cells were processed for immunoelectron microscopy using a modified Tokuyasu method [40] as described previously [41]. Briefly, pelleted cells were fixed overnight at 4°C in PBS buffered 4% paraformaldehyde containing 5% sucrose and 100 mM HEPES, and infiltrated with PBS containing 2.1 M sucrose over ∼10 hours, with repeated solution changes. Fixed cells were transferred to an aluminum cryosectioning stub (Ted Pella, Inc., Redding, CA) and immediately frozen in liquid nitrogen. Semi-thin (90 nm) cryosections were cut at −110°C with an UltraCut UCT/FCS cryomicrotome (Leica), using a diamond knife (Diatome US) and transferred to a Formvar-coated, carbon-coated, glow-discharged 100-mesh copper-rhodium EM grid. Following blocking of non-specific antibody binding sites with 10% calf serum in PBS, the sections were labeled by sequential incubation with rabbit antibodies to the N-terminal domain of Plin2 [36] and colloidal gold conjugated secondary antibodies (Ted Pella Inc., Redding, CA) and then negatively stained and embedded with 1% uranyl acetate, 1% methylcellulose in distilled water. Samples were viewed in a Tecnai TF20 electron microscope (FEI) operating at 200 KeV and images collected digitally.

### Statistical Analysis

Calculations were performed by using Prism 5.0 (GraphPad Software) and Microsoft Excel (Windows 2010, Microsoft). For each variable, 2 to 6 independent experiments were carried out. Differences between diet groups were tested for significance using an unpaired Student *t* test. Differences were considered significant at *P*≤0.05.

### Bioinformatic Analysis

STRING 9.0 (http://string.embl.de/), Gene ontology database (http://geneontology.org/), and KEGG (http://www.genome.jp/kegg) were used for protein interaction, biological function, and pathway analysis respectively.

## Results

### Diet effects on metabolism and hepatic lipid storage

**Figure 1:**
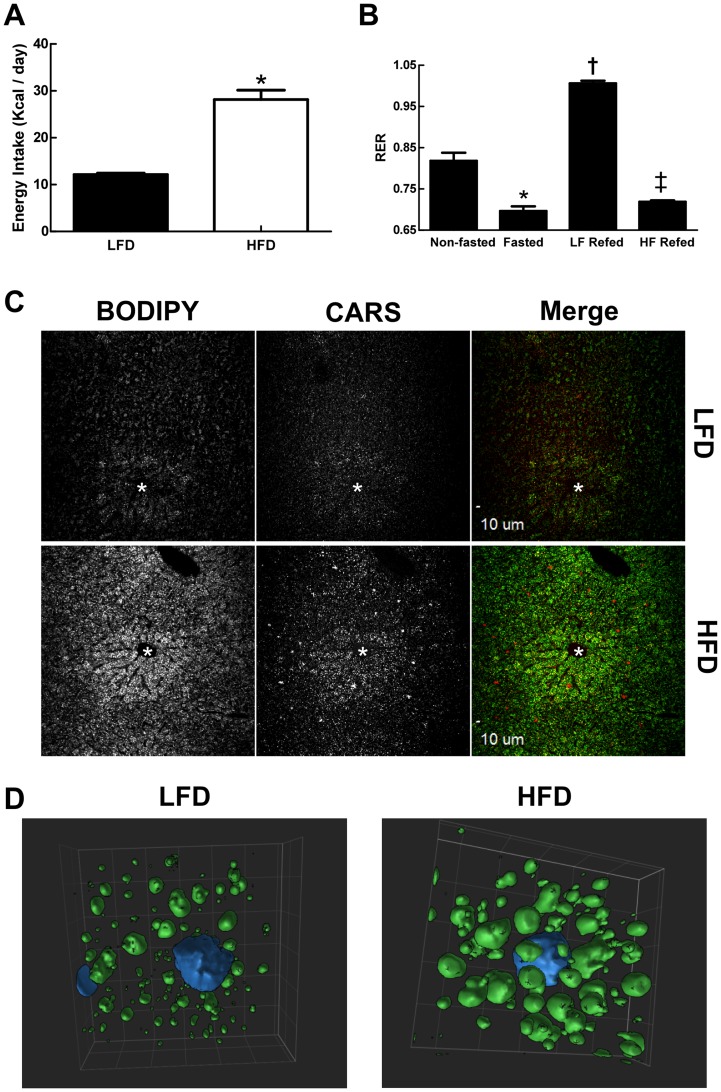
Diet effects on metabolism and hepatic lipid storage. (A) Effects of LF- and HF-refeeding on energy intake in fasted male mice. Values are means (± SD) for 4 animals in each group. Asterisk indicates HFD values differ from LFD values (p<0.01). (B) Energy usage in non-fasted, fasted and LF- and HF-refed male mice as determined by RER. Non-fasted and fasted values correspond to averages (± SD) of 8 animals obtained prior to refeeding. LF- and HF-refeeding values correspond to averages (± SD) for 4 animals in each group. Asterisk indicates values differ from non-fasted controls (p<0.001); dagger indicates values differ from non-fasted and fasted values (p<0.001); double dagger indicates values differ from LF refed and non-fasted values (p<0.001). (C) Representative images of frozen liver sections from LF- and HF-refed male stained with BODIPY and imaged by laser confocal (BODIPY) and CARS microscopy (200X magnification). Asterisks indicate central veins. (D) Representative surface-view of 3D projection images of single cells within liver sections from LF (LFD)- and HF (HFD) -refed mice obtained at 600X magnification. Nuclei are shown in blue.

Fasting and diet composition are known to influence food intake and liver metabolism, which in turn can affect hepatic lipid storage [42], [43]. Therefore, to define the effects of LF and HF diets on hepatic CLD properties, it was necessary first to establish the effects of these diets on energy intake and metabolism of fasted animals. Figure 1A shows that energy consumption of fasted animals that were refed the HF diet was significantly greater than that of animals refed the LF diet. We also found differences in the fuel utilization properties of LF- and HF-refed animals. Figure 1B shows the respiratory exchange ratio (RER) values of mice prior to fasting, following a 24 hr period of fast, and after refeeding with LF- or HF-diets. Prior to fasting, RER values were approximately 0.8, as fuel use reflected the broad mixture of carbohydrate, fat, and protein in the diet. During fasting, RER values dropped to approximately 0.7, indicating a switch to fat as the primary source of energy. Refeeding on a LF diet resulted in RER values that were close to 1, reflecting the preferential use of carbohydrate for energy production and the likelihood that *de novo* lipogenesis was induced [37]. In contrast, mice refed the HF diet had RER values that remained closer to 0.7, which indicated they were oxidizing fat for energy, like fasted animals.

The distribution and properties of CLD in liver sections of LF- and HF-refed mice were visualized by coherent anti-Stokes Raman scattering (CARS) microscopy [44], and by confocal laser microscopy after staining neutral lipids with BODIPY (493/503) (Figure 1C). CLD were detected throughout the liver in both LF- and HF-refed animals, although both CARS and BODIPY intensities appeared to be greater in the central vein region (zone 3). Consistent with the elevated fat content of HF diets, we found that CARS and BODIPY intensities of livers of HF-refed animals were greater than those of LF-refed mice. CLD properties were further defined by 3D laser confocal microscopy of BODIPY stained liver sections. Representative 3D projection images of CLD from central vein regions of hepatocytes of LF- and HF-refed mice (Figure 1D), indicate that CLD are larger, and appear to be more numerous, in animals refed the HF diet relative to those refed the LF diet.

### Diet effects on hepatic CLD protein composition

**Figure 2:**
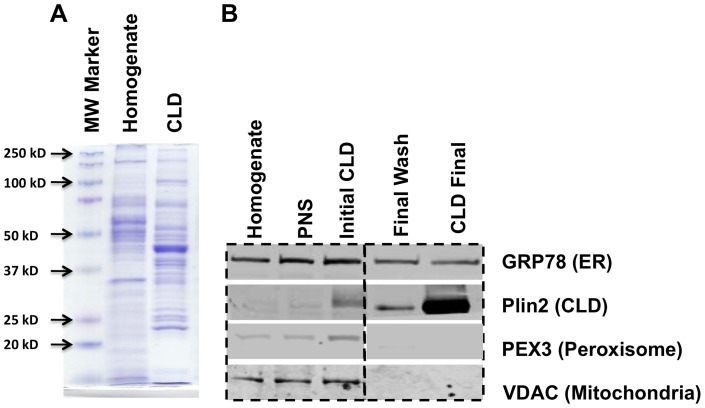
Unique protein patterns of isolated hepatic CLD. A) Coomassie blue staining profiles of proteins in liver homogenates and isolated CLD. B) Immunoblot analyses of equal amounts (25μg) of protein from liver homogenate, PNS, initial CLD, final CLD wash, and final enriched CLD fractions reacted with antibodies to GRP78, Plin2, PEX3, and VDAC.

To determine if LF- and HF-CLD differed in their protein compositions, CLD were isolated from liver extracts by multiple rounds of floatation [21]. We have previously documented that CLD isolated by this procedure are free of membrane structures and other organelles [21]. Figure 2A shows that the CLD fraction is enriched in specific protein bands that differ significantly from those present in the starting homogenate. The relative purity of the CLD-enriched fraction was estimated by probing the homogenate, post nuclear supernatant (PNS), the initial CLD fraction, the 3^rd^ (final) wash and final CLD fractions with antibodies to endoplasmic reticulum (GRP78), CLD (Plin2), peroxisome (PEX3), and mitochondria (VDAC) proteins. Figure 2B shows that Plin2 levels in the final CLD fraction were dramatically enriched over its levels in the starting homogenate and the initial CLD faction. We did not detect VDAC or PEX3 in the final CLD or in the final CLD wash fractions by immunoblot (Figure 2B) or by mass spectrometry analyses (see below), although both proteins were found in the homogenate and in initial CLD isolates. These results suggest that the final CLD fraction lacked significant mitochondria or peroxisome contamination, and that contaminating proteins in the initial CLD isolate were effectively removed by our floatation wash procedures. In contrast, and consistent with earlier proteomic evidence from other tissues and cell types [17], [45], we detected significant amounts GRP78 in the final CLD isolate. However, unlike fastidious CLD proteins such as Plin2, the relative levels GRP78 were not enriched in the final CLD isolate compared to the initial isolate and we continued to detect significant amounts of GRP78 in sucrose floatation wash fractions (Figure 2B). These observations indicate that GRP78 retains the ability to associate with hepatic CLD during their isolation; although its partial dissociation with each wash step suggests that the strength of this association is weaker than that of Plin2.

### Liver Specific- and Common-CLD Associated Proteins

We used LC-MS/MS analysis of trypsin digests of CLD protein extracts as a non-biased “shotgun” approach for identifying the protein compositions of LF- and HF-CLD. Only those proteins with two or more unique peptides, and that were found in repeat analyses of isolated CLD from duplicate fasting-refeeding experiments, were accepted as valid identifications. Overall, we identified 125 proteins on CLD from mice refed the LF diet, and 134 proteins on CLD from mice refed the HF diet. The identified proteins, their biological functions according to Uniprot and GO database descriptions, and estimates of their relative abundance as determined by their percentage of total spectra, are shown in the Table S2.

**Figure 3:**
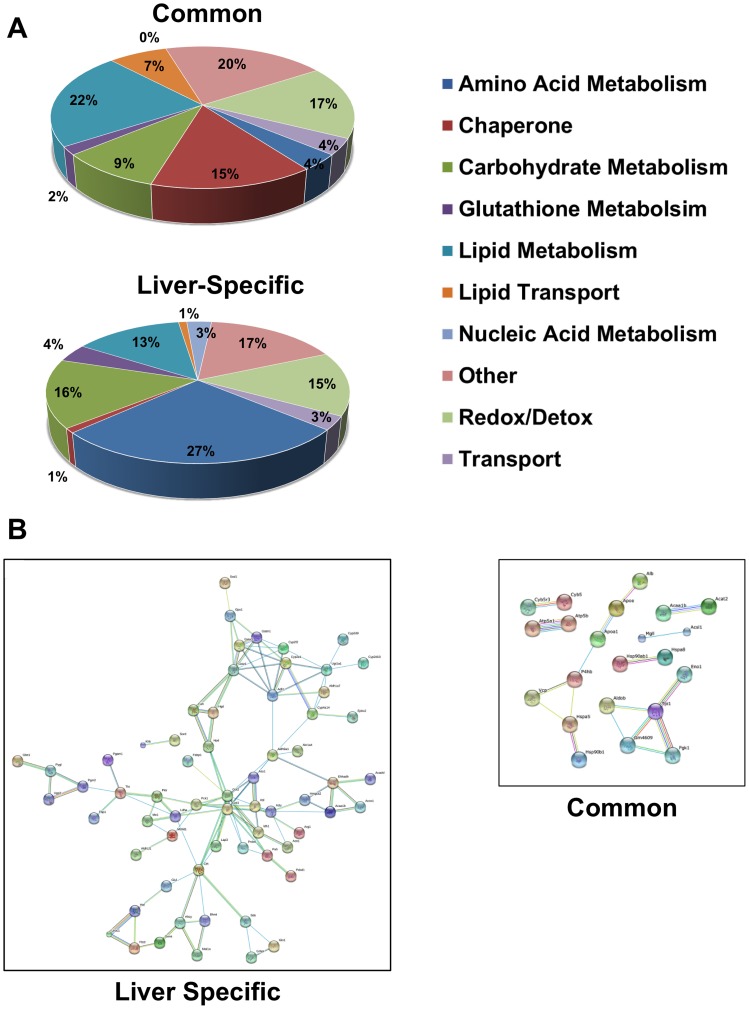
Hepatic CLD differs from other core CLD proteomes. (A) Functional categories of common- and liver-specific proteins categorized according to gene ontology (GO) annotations. (B) Association networks of common- and liver-specific CLD associated proteins predicted by the STRING 9.0 program with a confidence level of 0.8. Network edges represent predicted functional associations with different line colors standing for various types of evidence used in establishing the level of confidence. Red, fusion evidence; green, neighborhood evidence; blue, co-occurrence evidence; purple, experimental evidence; yellow, text-mining evidence; black, co-expression evidence. Non-network proteins are not shown.

Of the proteins found on either LF- or HF-CLD, 54 (36%) were identified previously on CLD from other mammalian sources (Table S3; the citations for these proteins are given in Table S2), and thus appear to represent common CLD associated proteins. Whereas, 98 proteins (64%) were not previously detected on CLD, and therefore potentially represent liver specific CLD associated proteins (Table S4). The functional categories of the common- and liver-specific CLD associated proteins are shown in Figure 3. Proteins involved in lipid metabolism (22%), redox/detoxification (17%) and chaperone functions (15%) accounted for over half of the common-CLD associated proteins, whereas enzymes of amino acid (27%) and carbohydrate metabolism (16%), and redox/detoxification (15%) pathways made up the majority of the liver-specific CLD associated proteins. We next used the STRING 9.0 program to examine the extent to which the identified proteins exhibited direct functional connections. As shown in Figure 3B, liver-specific proteins are organized into a series of discrete, interacting nodes, suggesting the existence of multiple functional linkages between nodes, and between proteins within a given node. Common-CLD associated proteins on the other hand appear to be less functionally related. We found that these proteins comprised only two non-interacting nodes; one related to protein processing, and another related to glycolysis.

We used the KEGG pathway database to further define potential functional interactions among common- and liver-specific CLD associated proteins (Table 1). We identified significant enrichment of common-CLD associated proteins in 9 KEGG pathways using a false discovery rate (FDR) value of p<0.05. The majority of the pathways are related to carbohydrate (4) and fatty acid metabolism (3), the others were related to amino acid metabolism (1) and protein processing (1). For liver-specific CLD associated proteins, we found significant enrichment in 26 KEGG pathway categories. Of these, 12 were related to amino acid metabolism, 6 were related to carbohydrate metabolism, 4 were related to fatty acid metabolism, 3 were related to xenobiotic metabolism and 1 was related to glutathione metabolism. Among the identified enzymes, several corresponded to large portions of the glycolysis/gluconeogenesis and cysteine/methonine pathways.

**Table 1:** Liver Specific- and Common-CLD Protein KEGG Pathways.

*Liver Specific CLD associated proteins*				
KEGG ID	Pathway	Number Of Genes	P-value fdr	
mmu00270	Cysteine and methionine metabolism	8	8.54E-07	
mmu00350	Tyrosine metabolism	7	1.44E-05	
mmu00330	Arginine and proline metabolism	8	1.73E-05	
mmu00010	Glycolysis/Gluconeogenesis	9	2.25E-05	
mmu00620	Pyruvate metabolism	7	2.25E-05	
mmu00360	Phenylalanine metabolism	5	2.26E-05	
mmu00071	Fatty acid metabolism	7	2.26E-05	
mmu00250	Alanine, aspartate and glutamate metabolism	6	3.61E-05	
mmu00982	Drug metabolism – cytochrome P450	8	9.24E-05	
mmu03320	PPAR signaling pathway	8	9.24E-05	
mmu00400	Phenylalanine, tyrosine and tryptophan biosynthesis	3	1.37E-04	
mmu00980	Metabolism of xenobiotics by cytochrome P450	7	3.10E-04	
mmu00480	Glutathione metabolism	6	6.03E-04	
mmu04146	Peroxisome	7	6.03E-04	
mmu00500	Starch and sucrose metabolism	5	9.90E-04	
mmu00450	Selenoamino acid metabolism	4	2.10E-03	
mmu00020	Citrate cycle (TCA cycle)	4	6.50E-03	
mmu00260	Glycine, serine and threonine metabolism	4	7.84E-03	
mmu00630	Glyoxylate and dicarboxylate metabolism	3	1.20E-02	
mmu00410	beta-Alanine metabolism	3	2.05E-02	
mmu00910	Nitrogen metabolism	3	2.05E-02	
mmu00280	Valine, leucine and isoleucine degradation	4	2.45E-02	
mmu00340	Histidine metabolism	3	2.78E-02	
mmu00030	Pentose phosphate pathway	3	4.16E-02	
mmu00903	Limonene and pinene degradation	2	4.24E-02	
mmu00640	Propanoate metabolism	3	4.24E-02	
***Common CLD associated proteins***				
mmu04146	Peroxisome	6		8.95E-04
mmu00010	Glycolysis/Gluconeogenesis	6		8.95E-04
mmu00071	Fatty acid metabolism	4		9.00E-03
mmu03320	PPAR signaling pathway	4		4.64E-02
mmu00051	Fructose and mannose metabolism	3		4.64E-02
mmu00650	Butanoate metabolism	3		4.64E-02
mmu00380	Tryptophan metabolism	3		4.64E-02
mmu00620	Pyruvate metabolism	3		4.64E-02
mmu04612	Antigen processing and presentation	4		4.64E-02

### Low- and High- Fat Specific CLD associated proteins

**Figure 4:**
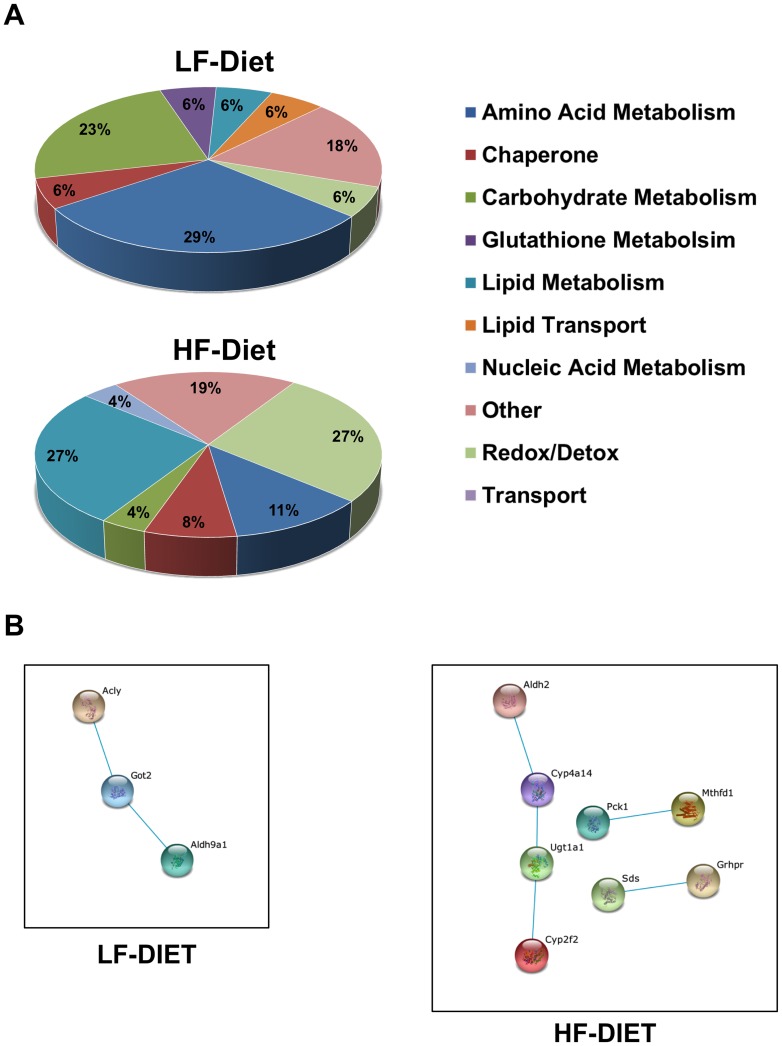
HFD induces expression of proteins from different pathways. (A) Functional categories of LF- and HF-specific CLD associated proteins categorized according to gene ontology (GO) annotations. (B) Association networks of LF- and HF-specific CLD associated proteins predicted by the STRING 9.0 program with a confidence level of 0.8. Color codes are as described in the legend to Figure 3.

The functional classes of proteins that were uniquely associated with LF- and HF-CLD exhibited distinct patterns (Figure 4A). The majority of the proteins uniquely associated with LF-CLD are involved in amino acid (29%) and carbohydrate (23%) metabolism. Whereas, most of the uniquely associated proteins on HF-CLD are related to lipid metabolism and redox/detoxifcation processes. Using the STRING 9.0 program to probe for functionally interactions between LF- and HF-CLD specific proteins, we found that LF-CLD specific proteins formed a single high stringency interaction node connecting enzymes involved in amino acid and acetate metabolism. Whereas HF-CLD specific proteins formed a high stringency node related to redox/detoxification processes, and two sets of individual connections between pyruvate and carbohydrate metabolism, and between methione/cysteine and dicarboxylic acid metabolism (Figure 4B).

Among the 17 proteins specifically found on LF-CLD, we did not detect significant enrichment in any KEGG pathway. However for the 26 proteins specifically found on HF-CLD, there was enrichment in 5 KEGG pathways with FDR values <0.05; PPAR signaling, ascorbate metabolism, pyruvate metabolism, fatty acid metabolism and glyoxylate metabolism. The identities of the HF-CLD associated proteins found in enriched KEGG pathways are shown in Table 2.

**Table 2:** Specific HFD CLD Protein KEGG Pathways.

KEGG ID	Pathway	P-value (fdr)	Gene
mmu00053	Ascorbate and aldarate metabolism	2.13E-03	Gulo
			Aldh2
			Ugt1a1
mmu03320	PPAR signaling pathway	7.47E-03	Apoa1
			Hmgcs2
			Pck1
			Cyp4a14
mmu00620	Pyruvate metabolism	1.37E-02	Grhpr
			Pck1
			Aldh2
mmu00071	Fatty acid metabolism	1.37E-02	Acadvl
			Aldh2
			Cyp4a14
mmu00630	Glyoxylate and dicarboxylate metabolism	4.73E-02	Mthfd1
			Grhpr

### Diet affects Plin2 CLD levels

**Figure 5:**
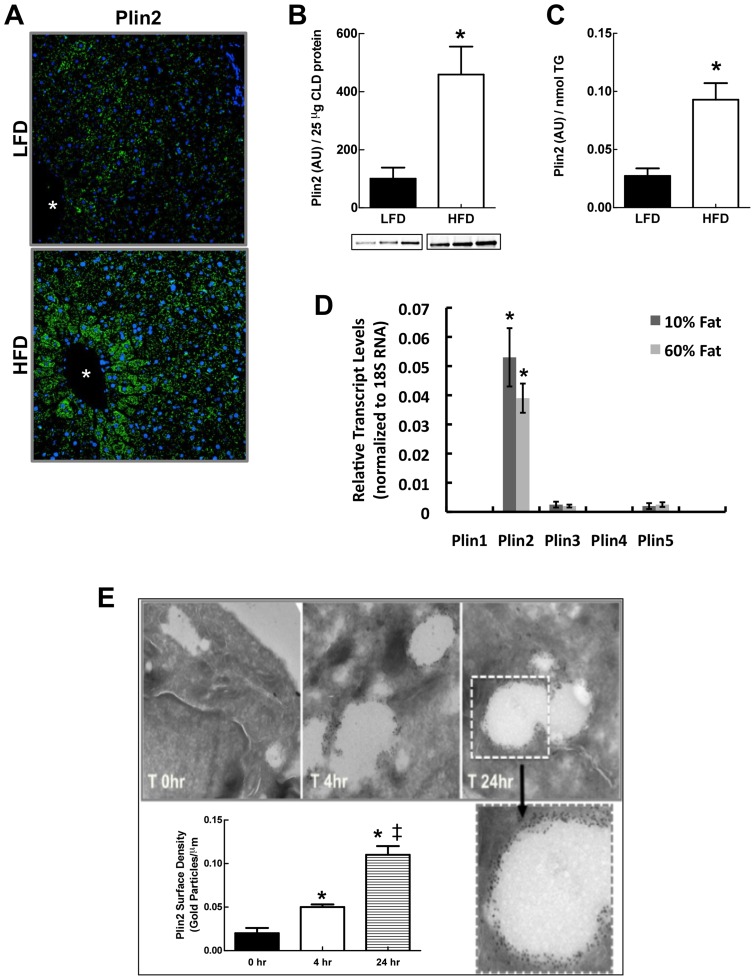
Diet effects Plin2 on CLD. (A) Representative confocal Plin2 immunofluorescence (green) images of liver sections from fasted male mice refed with LF (LFD) – and HF (HFD)-diets and stained with antibodies to Plin2. Nuclei (blue) were stained with DAPI. Asterisks indicate central veins. (B) Quantitative immunoblot analysis of Plin2 levels in enriched CLD protein extracts from fast-fed male mice on LF (LFD)- or HF(HFD)-diets. Insets show immunoblots of 25 μg of CLD protein from 3 mice. The graph shows the average (± SD) Plin2 levels normalized to 25 μg of total CLD protein from LF (N = 3) and HF (N = 3) mice. (C) CLD Plin2 levels normalized to CLD TG content. Values are means (± SD) for LF (N = 3) and HF (N = 3) refed animals. Asterisks in B and C indicate HFD values differ from LFD values (p<0.0001). (D) Transcript levels of PLIN family members in livers of fasted and refed mice on LFD and HFD quantified by qRT-PCR using primers listed in Table S1. Values are means± SD normalized to 18S RNA. Asterisks indicate Plin2 transcript levels are significantly elevated over transcript levels for Plins 1,3,4, and 5. (E) Effects of HF feeding on Plin2 surface density in HEK293 cells stably expressing Plin2-VSV. Images are representative electron micrographs of anti-PLIN2-gold particle labeled cells that were cultured in oleic acid-supplemented media for 0h, 4h or 24h. An enlarged micrograph of a CLD at 24h is shown. Average (± SD) Plin2 surface densities on CLD at each time point are shown for 50–75 CLD from triplicate cultures. The experiment was repeated twice with similar results. Asterisks indicate statistically significant differences from T = 0 time point, double dagger indicates statistically significant differences between 4h and 24h time points.

In addition to finding qualitative differences in the protein compositions of LF- and HF-CLD, we found that LF and HF diets appeared to influence the relative abundance of some CLD-associated proteins, as suggested by differences in total spectra percentages (Table S2). One of these proteins was the CLD scaffolding protein, Plin2. To determine if spectra percentage differences observed for Plin 2 on LF- and HF-CLD reflected actual abundance differences, we investigated the effects of LF and HF-refeeding on hepatic Plin2 by confocal immunofluorescence (IF) microscopy, and by quantitative immunoblot analysis of isolated CLD (Figure 5). IF microscopy showed significant Plin2 immunostaining in livers of both LF- and HF-refed mice, with HF-refed mice exhibiting greater Plin2 staining intensities than LF-refed mice, particularly within central vein regions (Figure 5A). Quantitative immunoblot analyses showed that when compared to total CLD protein (Figure 5B), or to total CLD triglyceride (TG) levels (Figure 5C), HF-refeeding increased the average amount of Plin2 on CLD by approximately 4-fold over that found on CLD from LF-refed animals. HF refeeding did not increase the total amount of protein per unit of CLD TG (data not shown), which suggests that the enrichment of Plin2 on HF-CLD was not associated with a generalized increase in the protein content of CLD from HF-refed animals.

Fasting and HF diets are known to increase hepatic Plin2 transcript levels [46]. Thus, we were interested in determining whether differences in the relative abundance of Plin2 on LF- and HF-CLD reflected differences in the effects of LF and HF diets on hepatic Plin2 expression. As shown in Figure 5D, we found that Plin2 transcript levels in total hepatic RNA were similar for HF- and LF-refed mice. The data in Figure 5D also show that hepatic Plin2 transcript levels are several fold greater than those of other PLIN family members, and that hepatic expression levels of other PLIN family genes also were not influenced by the content of fat in the refeeding diet. Collectively, these data indicate that Plin2 is the most abundantly expressed member of the PLIN family in livers of fasted and refed mice, and that differences in the effects of LF- and HF-refeeding on CLD levels of Plin2 are unrelated to the effects of these diets on Plin2 expression.

### High fat feeding increases Plin2 surface density on CLD

As a CLD surface associated protein, an increase in the relative amount of Plin2 on CLD could be due to loss of other CLD-associated proteins, or to absolute increases in its surface density. To address this issue, we quantified the effects of HF exposure on the CLD surface density of Plin2 by electron microscopy after labeling Plin2 with immuno-gold particles. For these experiments, we used Plin2 negative HEK293 cells that constitutively expresses recombinant mouse Plin2 under control of the CMV promoter [47] to avoid potential effects of fat exposure on Plin2 expression. Figure 5E shows that CLD in these cells increased in size following feeding with 100 μM oleic acid (OA), and that the number of anti-Plin2 conjugated-gold particles on the surface of individual CLD increased as a function of time in OA-supplemented media. The average CLD surface densities of Plin2 in cells incubated in control media without OA supplementation (T = 0 hr), or in cells supplemented with OA for 4 or 24 hrs are shown in the graph in Figure 5E. Plin2 surface densities in cells incubated in OA-supplemented medium were about 2-fold greater than their T0 values after 4 hrs (p<0.01), and approximately 6-fold greater after 24 hrs (p<0.001). These data provide direct evidence that the surface density of Plin2 on CLD is dynamically regulated and increased under conditions of high fat exposure.

### Endoplasmic Reticulum (ER) Chaperone Proteins Localize to Hepatic CLD

**Figure 6:**
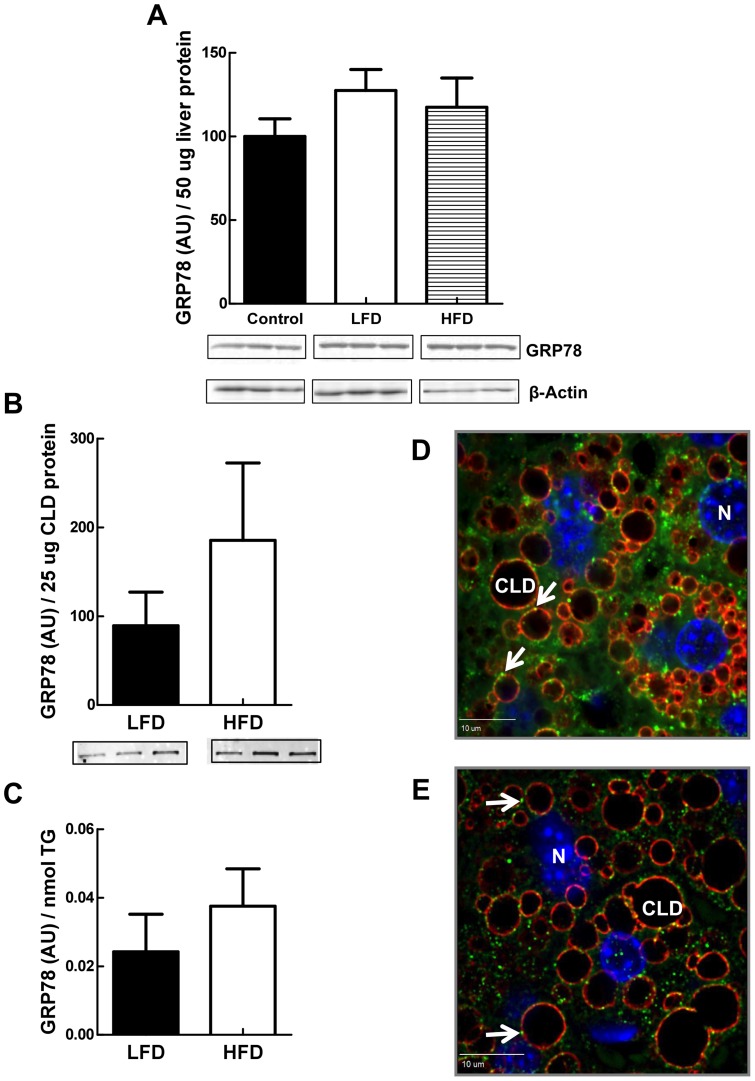
ER proteins are associated with CLD. (A) Quantitative immunoblot analysis of GRP78 levels in liver extracts from non-fasted male mice (Control) and fast-fed male mice on LF- or HF-diets. Insets show immunoblots of 50 μg of total liver homogenate protein from 3 mice probed with antibodies to GRP78 or β-actin. The graph shows the average (± SD) GRP78 levels normalized to β-actin from Control (3), LF- (LFD) (N = 3) and HF- (HFD) (N = 3) refed mice. (B) Quantitative immunoblot analysis of GRP78 levels in enriched CLD protein extracts from fast-fed male mice on LF- or HF-diets. Insets show immunoblots of 25 μg of CLD protein from 3 mice. The graph shows the average (± SD) GRP78 levels normalized to 25 μg of CLD protein from LF- (LFD) (N = 3) and HF- (HFD) (N = 3) refed mice. (C) CLD GRP78 levels normalized to CLD TG content. Values are means (± SD) for LFD (N = 3) and HFD (N = 3) refed animals. (D) Representative confocal immunofluorescence images of liver sections from HF refed mice stained with antibodies to Plin2 (red) and GRP78 (green). (E) Representative confocal immunofluorescence images of liver sections from HF refed mice stained with antibodies to Plin2 (red) and PDI (green). Nuclei in images in D and E (blue) were stained with DAPI. Arrows in D and E indicate localization of GRP78 or PDI on CLD. Bar is 10 μm.

ER proteins, including several with chaperone function, have been identified on isolated CLD from various mammalian cells and tissues [21], [22], [24], [26], [48], as well as from drosophila larvae [19]. We detected GRP78 in highly enriched hepatic CLD by immunoblot analysis (Figure 2), and we found GRP78 and numerous other chaperone-related proteins on LF- and HF-CLD by proteomic analysis (Table S2). Hepatic GRP78 expression is upregulated in response to ER stress [49], and elements of the ER stress pathway are known to play crucial roles in regulating hepatic lipid metabolism, including lipogenesis [50]. To assess possible LF- and HF-diet refeeding effects on chaperone-CLD interactions in the liver, and better understand the nature of these interactions, we quantified CLD-GRP78 levels in response to LF- and HF-refeeding, and directly investigated the association of GRP78 with CLD in hepatic tissue by IF analysis (Figure 6). The levels of GRP78 in liver homogenates of LF- and HF-refed mice did not differ significantly from each other, or from GRP78 levels found in livers of non-fasted mice, which suggests that hepatic CLD responses are not associated with obvious ER stress. Furthermore, we did not find significant differences in the amount of CLD-associated GRP78 in LF- and HF-refed livers, demonstrating that unlike Plin2, CLD levels of GRP78 are not influenced by diet.

Evidence of direct association between GRP78 and CLD in intact cells has been obtained in adipocytes [51]. To verify that GRP78 directly associates with CLD in intact hepatocytes, we visualized liver sections from fasted mice that were refed with the HF diet and immunostained for Plin2 and GRP78 with laser confocal microscopy (Figure 6D). GRP78 immunostaining was detected in the ER network of hepatocytes and on the surface of their CLD. In contrast to the relatively uniform staining intensity of Plin2 on CLD, GRP78 localized as discrete patches on the CLD surface, in a pattern similar to that described for CLD in adipocytes [51].

Protein disulfide isomerase (PDI) is another prominent ER chaperone protein that was identified by proteomic analysis of hepatic CLD from LF-and HF-refed mice (Table S2). We validated the association of PDI with isolated CLD by immunoblot analysis (data not shown), and confirmed its CLD localization by laser confocal imaging of immunostained stained liver sections (Figure 6E). Similar to GRP78, PDI localized as discrete patches on the surface of Plin2-positive CLD. Collectively, the GRP78 and PDI immunostaining data validate proteomic evidence of ER chaperone protein-CLD association and suggest that chaperones exhibit distinct organizational patterns on the CLD surface.

### LF- and HF-refeeding differentially affect CLD levels of the methionine-metabolizing enzyme BHMT

**Figure 7:**
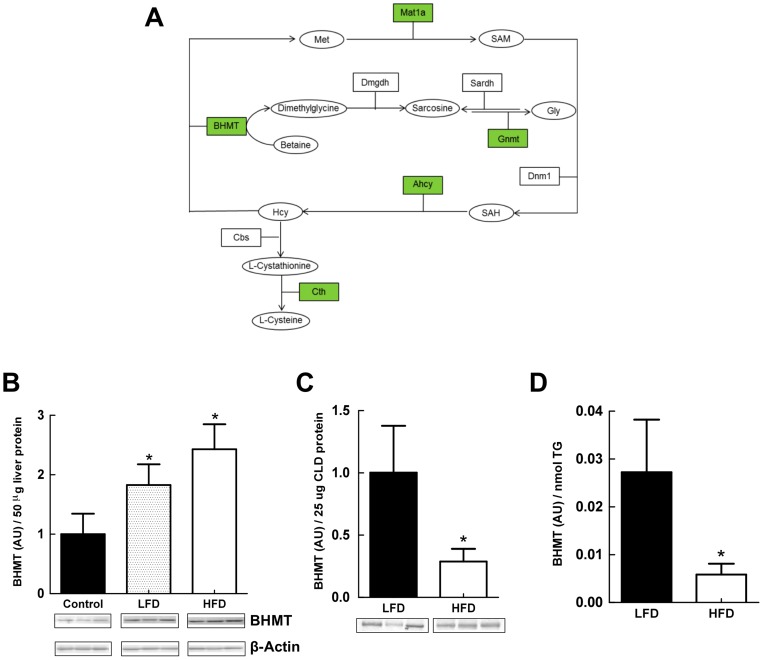
Methionine-Cysteine pathway proteins associated with CLD. (A) Schematic diagram representing the methionine/cysteine pathway. Boxes represent proteins, circles represent metabolites. Green colored boxes indicate proteins identified on hepatic CLD. (B) Quantitative immunoblot analysis of BHMT levels in liver extracts from non-fasted male mice (Control) and refed male mice on LF- or HF-diets. Insets show immunoblots of 50 μg of total liver homogenate protein from 3 mice probed with antibodies to BHMT or β-actin. The graph shows the average (± SD) BHMT levels normalized to β-actin from Control (3), LFD (N = 3) and HFD (N = 3) refed mice. Asterisks indicate LFD and HFD values differ from Control values (p<0.005). (C) Quantitative immunoblot analysis of BHMT levels in CLD protein extracts from refed male mice on LF- or HF-diets. Insets show immunoblots of 25 μg of CLD protein from 3 mice. The graph shows the average (± SD) BHMT levels normalized to 25 μg of CLD protein from LFD (N = 3) and HFD (N = 3) mice. (D) CLD BHMT levels normalized to CLD TG content. Values are means (± SD) for LFD (N = 3) and HFD (N = 3) refed animals. Asterisks in C and D indicate HFD values differ from LFD values (p<0.001).

Pathway analysis, revealed significant enrichment of enzymes associated with the cysteine-homocysteine-methionine pathway on liver-CLD (Table 1). Figure 7A, shows the relationship between proteins identified by mass spectrometry and specific steps of the cysteine-methionine metabolism pathway. Betaine homocysteine S-methlytransferase (BHMT), a critical regulatory enzyme of this pathway [52], is an abundant liver protein and one of the top proteomic hits on CLD from LF- and HF-fed mice. In mice, loss of BHMT has been shown to induce hepatosteatosis [53], and high fat feeding has been shown to increase hepatic BHMT transcript levels [54]. We were thus interested in determining if diet affected the amount of BHMT associated with CLD. Figure 7B shows that BHMT levels in whole liver extracts from LF- and HF-refed animals were significantly (77% and 128% respectively) higher than those of non-fasted control animals. Although hepatic BHMT levels in animals refed the HF-diet tended to be higher than those of LF-refed animals, the differences did not reach statistical significance. In contrast, levels of BHMT associated with LF-CLD were about 3-times higher than those associated with HF-CLD when normalized to either total CLD protein, or to CLD-TG content (Figure 7 C and D). These results provide direct evidence for the presence of BHMT on CLD, and demonstrate that its CLD association is altered by diet.

## Discussion

Neutral lipid accumulation in the liver, a critical determinant of hepatic lipid homeostasis and liver health [3], [34], [55], is affected by diet and alterations in metabolic function [56], [57]. CLD are responsible for storage and mobilization of neutral lipid stores through the actions of specific surface associated proteins. Although earlier studies have identified proteins associated with hepatic CLD from mice [21], [29], the data presented here provide the first comprehensive non-biased description of the mouse hepatic CLD proteome. The novel findings of this study are that the hepatic CLD protein composition appears to be distinct from that of CLD from other sources; that enzymes of multiple metabolic pathways are associated with hepatic CLD; and that the protein composition of hepatic CLD from fasted and refed mice is qualitatively and quantitatively influenced by dietary fat content, and corresponds to alterations in hepatic metabolic properties. Together, these findings provide evidence that CLD properties are dynamically regulated by the metabolic status of the liver, and that CLD may function in coordinating diverse metabolic activities within liver cells.

The proteins identified in our study were considered to be CLD associated based on their ability to remain bound to CLD through four rounds of floatation isolation. It is possible that using this criterion that at least some of the identified proteins may represent adventitious associations with CLD, especially in the case of low abundance proteins. However, several lines of evidence from our study suggest that the identified proteins are present on CLD as a result of bone fide interactions. First, as shown for VDAC and PEX3, the isolation/wash procedures effectively remove contaminating proteins to levels below that detectable by mass spectrometry. Second, the consistent effects of diet on CLD protein composition cannot be readily explained by simple contamination. Third, the relative abundance of cytoplasmic proteins, such as BHMT, in the final CLD fraction does not correlate with their hepatic levels, as would be expected if their association with CLD was due to adventitious binding. Fourth, proteins such as GRP78 and PDI that have been assumed to represent ER contamination of CLD [20], have been shown to be in close contact with the CLD surface in intact tissues by confocal immunofluoresence microscopy ([51] and Figure 6). Corroboration of the CLD associations of identified proteins will require additional high resolution imaging of hepatic tissue. Alternative methods such as heavy isotope labeling approaches that have been successfully used for proteomic quantification in cultured cells are not practical for *in vivo* studies [20], particularly for studies involving diet and metabolism in which precursor pools and protein isotope enrichments can be affected [58]. In addition, concepts about CLD protein composition based on biochemical enrichment correlations [20], potentially overlook weaker, mass action driven interactions, that can occur *in situ,* and possibly exclude interactions that occur with proteins from other cellular compartments [59].

### Metabolic functions of hepatic CLD

The primary biological function of CLD is generally understood to be neutral lipid storage, which is thought to involve the integrated actions of ER enzymes and specific CLD-associated proteins [60]. In agreement with this concept, multiple proteomic studies have consistently detected various ER proteins, lipid metabolism enzymes, and members of the PLIN family of CLD-associated proteins on isolated CLD from a variety of mammalian cells and tissues [21]–[31], [48], [61]. There is also growing evidence that CLD may sequester proteins, thereby indirectly contributing to other cellular functions [45]. Our study provides evidence that, at least within the liver, CLD may also function as a platform for coordinating metabolic functions by bringing together elements of specific metabolic pathways. Although additional work is needed to formally establish this concept, we found that the hepatic CLD protein composition is significantly enriched in enzymes composing KEGG pathways related to amino acid, carbohydrate, lipid and xenobiotic metabolism. Further, the identified proteins comprised multiple networks of functionally linked enzymes that, in some cases, correspond to intact portions of metabolic pathways.

### CLD properties reflect differences in liver metabolism

Our data indicate that the differential effects of LF- and HF-diet on hepatic CLD protein compositions reflect, in part, differences in hepatic metabolic properties. Indirect calorimetry measurements documented that LF- and HF-refeeding differentially affected the energy metabolism of fasted mice; inducing lipogenesis and the use of carbohydrates for fuel in LF-refed animals, while stimulating the use of fat for fuel in HF-refed animals. Consistent with these metabolic differences, we found a selective enrichment of enzymes involved in amino acid and carbohydrate metabolism, and *de novo* fatty acid synthesis on LF-CLD. In contrast, enzymes and proteins involved in fatty acid metabolism and lipid transport were enriched on HF-CLD. Additional studies are required to assess the functional importance of these differences. However, it is likely that protein composition differences will reflect subtle modulations of CLD activity, rather than overt changes in their function, since we did not detect large qualitative differences in the protein compositions of LF- and HF-CLD.

This concept is supported by observations that CLD binding of BHMT, a key enzyme in cysteine-methionine metabolism, is differentially affected by dietary fat content and metabolic status of the liver, and is independent of total tissue BHMT levels. In conjunction with evidence that multiple members of the cysteine-methionine metabolic pathway are present on hepatic CLD, the ability of diet to influence CLD-BHMT interactions raises the possibility that CLD may contribute to hepatic metabolic functions by providing a platform for coordinating enzymatic reactions associated with cysteine-methionine metabolism.

### Diet induces alterations in CLD surface organization

Diet effects on liver metabolic properties, including transcript expression and protein profiles, have been identified in both long-term and fasting-refeeding studies [32], [33], [62], [63]. There is also increasing recognition that diet influences the molecular properties of hepatic organelles, including mitochondria and ER [64], [65]. Our data expand the effects of diet to include hepatic CLD, documenting directly that the amount of dietary fat affects their molecular properties. The observed effects include alterations in CLD-associated levels of Plin2 and BHMT, both of which are functionally linked to fatty liver formation in mice [8], [53]. Plin2 is a CLD-scaffolding protein [10] that plays an essential role in regulating hepatic lipid accumulation [11], [12]. The finding that HF refeeding increases the CLD surface density of Plin2 provides evidence that its surface organization is dynamically regulated, and that alterations in the surface properties of Plin2 may contribute to its scaffolding and/or lipid storage functions. As yet, it is unclear how diet-induced changes in Plin2 surface density affect hepatic CLD properties. However, we have previously demonstrated that CLD size is decreased in mammary glands of Plin2-deficient mice compared to that of WT mice [66]. Thus, regulating the CLD surface density of Plin2 may be a mechanism for determining CLD size, which in the liver appears to be influenced by dietary fat content.

In summary, our study has described the mouse hepatic CLD proteome, and demonstrated that it is markedly different from proteomes of CLD from other cells and tissues. We have also shown that the hepatic CLD proteome is dynamically influenced by dietary fat content, and related to differences in liver metabolic properties. The proteins found on hepatic CLD are enriched in enzymes with extensive functional connections known to be important for liver metabolism. These findings are consistent with growing evidence that CLD protein compositions are influenced by cellular function, metabolic disorders and/or the physical properties of CLD [14], [15], [24], and they provide support for an expanded role for CLD in regulating cellular metabolic properties beyond that of lipid storage.

## Supporting Information

Table S1 QPCR Primers and Probes.
(DOCX)

Table S2 Liver CLD Proteome.
(DOCX)

Table S3 Common CLD Associated Proteins.
(DOCX)

Table S4 Liver Specific CLD Associated Proteins.
(DOCX)
