# Supplementary figures and images for: Dynamic Regulation of Hepatic Lipid Droplet Properties by Diet
Source: PLoS One. 2013 Jul 11;8(7):e67631. doi: 10.1371/journal.pone.0067631 (PMC3708958; doi:10.1371/journal.pone.0067631)

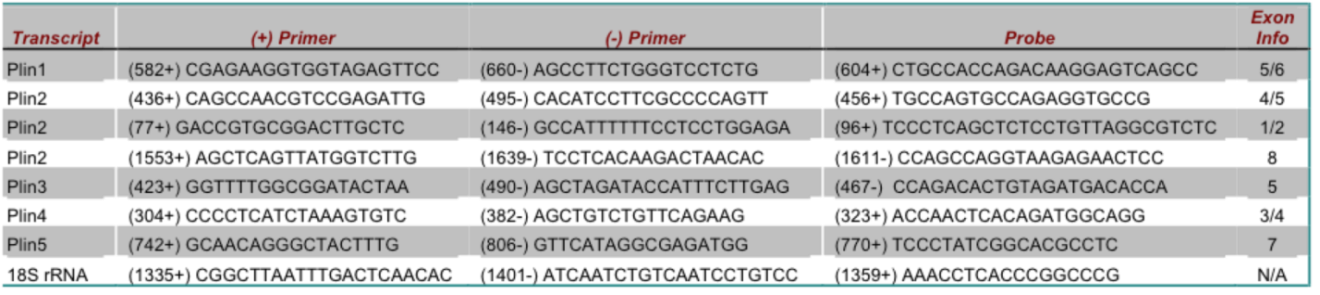


**Table S1. QPCR Primers and Probes**

Supplement: Table S1 — QPCR Primers and Probes. (DOCX) [file pone.0067631.s001.docx]
