# Supplementary material for: Dynamic Regulation of Hepatic Lipid Droplet Properties by Diet
Source: PLoS One. 2013 Jul 11;8(7):e67631. doi: 10.1371/journal.pone.0067631 (PMC3708958; doi:10.1371/journal.pone.0067631)
Supplement: Table S2 — Liver CLD Proteome. (DOCX) [file pone.0067631.s002.docx]

| **Table S2. Liver CLD Proteome** | | | | | |
| --- | --- | --- | --- | --- | --- |
|  | | | | |  |
|  | **Gene** | **Uniprot ID** | **LFD** | **HFD** | **Reference** |
| ***Amino Acid Metabolism  (GO:0006520)*** |  |  |  |  |  |
| Adenosyl-homocysteinase | Ahcy | P50247 | 0.121% | 0.080% |  |
| 4-trimethylamino-butyraldehyde dehydrogenase | Aldh9a1 | Q9JLJ2 | 0.015% |  |  |
| Arginase-1 | Arg1 | Q61176 | 0.140% | 0.144% |  |
| Argininosuccinate lyase | Asl | Q91YI0 | 0.094% | 0.094% |  |
| Argininosuccinate synthase | Ass1 | P16460 | 0.196% | 0.220% |  |
| Betaine--homocysteine  S-methyltransferase 1 | Bhmt | O35490 | 0.347% | 0.250% |  |
| Carbonic  anhydrase 3 | Ca3 | P16015 | 0.130% | 0.092% | [30] |
| Carbamoyl-phosphate synthase [ammonia], mitochondrial | Cps1 | Q8C196 | 0.041% | 0.140% | [28, 30] |
| Cystathionine gamma-lyase | Cth | Q8VCN5 | 0.054% | 0.055% |  |
| Dihydropyrimidinase | Dpys | Q9EQF5 | 0.009% |  |  |
| Fumarylacetoacetase | Fah | P35505 | 0.085% | 0.067% |  |
| Formimidoyltransferase cyclodeaminase | Ftcd | Q91XD4 | 0.048% | 0.043% |  |
| Glutamine synthetase | Glul | P15105 | 0.060% | 0.042% |  |
| Glycine N-methyltransferase | Gnmt | Q9QXF8 | 0.121% | 0.087% |  |
| Aspartate aminotransferase, cytoplasmic | Got1 | P05201 | 0.049% | 0.044% |  |
| Aspartate aminotransferase, mitochondrial | Got2 | P05202 | 0.005% |  |  |
| Alanine aminotransferase 1 | Gpt | Q8QZR5 |  | 0.009% |  |
| Maleylacetoacetate isomerase | Gstz1 | Q9WVL0 | 0.012% | 0.004% |  |
| Histidine ammonia-lyase | Hal | P35492 | 0.003% |  |  |
| Homogentisate 1,2-dioxygenase | Hgd | O09173 | 0.021% |  |  |
| 4-hydroxyphenylpyruvate dioxygenase | Hpd | P49429 | 0.039% | 0.042% |  |
| Cytosol aminopeptidase | Lap3 | Q9CPY7 | 0.009% | 0.019% |  |
| S-adenosylmethionine synthase isoform type-1 | Mat1a | Q91X83 | 0.099% | 0.106% |  |
| C-1-tetrahydrofolate synthase, cytoplasmic | Mthfd1 | Q922D8 |  | 0.007% |  |
| Phenylalanine 4 hydroxylase | Pah | P16331 | 0.028% | 0.049% |  |
| L-serine dehydratase/L-threonine deaminase | Sds | Q8VBT2 |  | 0.009% |  |
| Sepiapterin reductase | Spr | Q64105 | 0.006% | 0.004% |  |
| Urocanate hydratase | Uroc1 | Q8VC12 | 0.041% | 0.028% |  |
|  |  |  |  |  |  |
| ***Protein Metabolism (GO:0044267) Chaperones*** |  |  |  |  |  |
| Heat shock protein HSP 90-beta | Hsp90ab1 | P11499 | 0.072% | 0.041% | [24, 27, 32, 50, 62] |
| Endoplasmin | Hsp90b1 | P08113 |  | 0.009% | [22, 27, 29-32, 50, 62] |
| 78 kDa glucose-regulated protein | Hspa5 | P20029 | 0.022% | 0.108% | [23-27, 29-31, 50, 62] |
| Heat shock cognate 71 kDa protein | Hspa8 | P63017 | 0.093% | 0.072% | [27, 30, 32, 50, 62] |
| Protein disulfide-isomerase | P4hb | P09103 | 0.019% | 0.030% | [23, 24, 27, 30, 31, 50, 62] |
| Phenazine biosynthesis-like domain-containing protein 1 | Pbld1 | Q9DCG6 | 0.018% |  |  |
| Protein disulfide-isomerase A3 | Pdia3 | P27773 | 0.013% | 0.003% | [24, 29, 30, 50, 62] |
| Protein disulfide-isomerase A6 | Pdia6 | Q922R8 |  | 0.037% | [23, 24, 29, 30, 32, 50, 62] |
| Peptidyl-prolyl cis-trans isomerase A | Ppia | P17742 | 0.075% | 0.048% | [23, 24] |
|  |  |  |  |  |  |
| ***Carbohydrate Meatbolism (GO:0005975)*** |  |  |  |  |  |
| Cytoplasmic aconitate hydratase | Aco1 | P28271 | 0.016% | 0.018% |  |
| Fructose-bisphosphate aldolase B | Aldob | Q91Y97 | 0.250% | 0.193% | [50] |
| Pancreatic alpha-amylase | Amy2 | P00688 | 0.007% |  |  |
| Bifunctional ATP-dependent dihydroxyacetone kinase/FAD-AMP lyase (cyclizing) | Dak | Q8VC30 | 0.081% | 0.056% |  |
| Alpha-enolase | Eno1 | P17182 | 0.034% | 0.062% | [30, 50] |
| Fructose-1,6-bisphosphatase 1 | Fbp1 | Q9QXD6 | 0.130% | 0.142% |  |
| 1,4-alpha-glucan-branching enzyme | Gbe1 | Q9D6Y9 | 0.003% | 0.022% |  |
| Isocitrate dehydrogenase [NADP] cytoplasmic | Idh1 | O88844 | 0.051% | 0.042% |  |
| Ketohexokinase | Khk | P97328 | 0.009% |  |  |
| L-lactate dehydrogenase A chain | Ldha | P06151 | 0.020% | 0.033% |  |
| Malate dehydrogenase, cytoplasmic | Mdh1 | P14152 | 0.032% | 0.020% | [28, 30] |
| Phosphoglycerate mutase 1 | Pgam1 | Q9DBJ1 | 0.004% |  |  |
| Phosphoglycerate kinase 1 | Pgk1 | P09411 | 0.011% | 0.041% | [50] |
| Phosphoglucomutase-1 | Pgm1 | Q9D0F9 |  | 0.010% |  |
| Pyruvate kinase isozymes R/L | Pklr | P53657 | 0.028% | 0.009% |  |
| Glycogen phosphorylase, liver | Pygl | Q9ET01 | 0.034% | 0.012% |  |
| Sorbitol dehydrogenase | Sord | Q64442 | 0.082% | 0.098% |  |
| Transketolase | Tkt | P40142 | 0.014% | 0.009% |  |
| Triosephosphate isomerase | Tpi1 | P17751 | 0.026% | 0.032% | [62] |
| UTP--glucose-1-phosphate uridylyltransferase | Ugp2 | Q91ZJ5 | 0.022% |  |  |
|  |  |  |  |  |  |
| ***Glutathione Metabolism (GO:0006749)*** |  |  |  |  |  |
| Lactoylglutathione lyase | Glo1 | Q9CPU0 | 0.009% |  |  |
| Glutathione peroxidase 1 | Gpx1 | P11352 | 0.048% | 0.043% |  |
| Glutathione S-transferase A3 | Gsta3 | P30115 | 0.085% | 0.059% |  |
| Glutathione S-transferase Mu 1 | Gstm1 | P10649 | 0.120% | 0.109% |  |
| Glutathione S-transferase P 1 | Gstp1 | P19157 | 0.138% | 0.156% | [62] |
|  |  |  |  |  |  |
| ***Lipid Metabolism (GO:0006629)*** |  |  |  |  |  |
| ATP-binding cassette sub-family D member 3 | Abcd3 | P55096 |  | 0.005% |  |
| CGI58 | Abhd5 | Q9DBL9 |  | 0.008% | [22-27, 31, 50, 62] |
| 3-ketoacyl-CoA thiolase A, peroxisomal | Acaa1a | Q921H8 | 0.026% | 0.048% | [50] |
| 3-ketoacyl-CoA thiolase B, peroxisomal | Acaa1b | Q8VCH0 | 0.004% | 0.006% |  |
| Very long-chain specific acyl-CoA dehydrogenase | Acadvl | P50544 |  | 0.006% |  |
| Acetyl-CoA acetyltransferase, cytosolic | Acat2 | Q8CAY6 | 0.005% | 0.011% | [50] |
| ATP-citrate synthase | Acly | Q91V92 | 0.015% |  |  |
| Peroxisomal acyl-coenzyme A oxidase 1 | Acox1 | Q9R0H0 | 0.016% | 0.029% |  |
| Long-chain-fatty-acid--CoA ligase 1 | Acsl1 | P41216 | 0.022% | 0.045% | [22, 23, 30, 31] |
| Estradiol 17 beta-dehydrogenase 5 | Akr1c6 | P70694 | 0.055% | 0.035% | [32] |
| ATP synthase subunit alpha, mitochondrial | Atp5a1 | Q03265 |  | 0.023% | [30] |
| ATP synthase subunit beta, mitochondrial | Atp5b | P56480 | 0.003% | 0.024% | [30, 50] |
| Carboxylesterase 3 | Ces1d | Q8VCT4 | 0.032% | 0.059% | [30] |
| Cytochrome b5 | Cyb5a | P56395 | 0.007% | 0.022% | [30, 50] |
| NADH-cytochrome b5 reductase 3 | Cyb5r3 | Q9DCN2 | 0.018% | 0.080% | [23, 24, 26-29, 31, 50, 62] |
| Cytochrome P450 2E1 | Cyp2e1 | Q05421 | 0.010% | 0.011% |  |
| Peroxisomal bifunctional enzyme | Ehhadh | Q9DBM2 | 0.028% | 0.056% |  |
| Epoxide hydrolase 2 | Ephx2 | P34914 | 0.057% | 0.046% |  |
| Fatty acid synthase | Fasn | P19096 | 0.143% | 0.041% | [50, 62] |
| Hydroxymethylglutaryl-CoA synthase | Hmgcs2 | P54869 |  | 0.018% |  |
| Estradiol 17-beta-dehydrogenase 11 | Hsd17b11 | Q9EQ06 |  | 0.018% |  |
| Monoglyceride lipase | Mgll | O35678 | 0.015% | 0.034% | [28, 30, 31, 50] |
| Phosphoenolpyruvate carboxykinase, cytosolic [GTP] | Pck1 | Q9Z2V4 |  | 0.011% |  |
| Inorganic pyrophosphatase | Ppa1 | Q9D819 | 0.003% | 0.009% |  |
| Peroxiredoxin-6 | Prdx6 | O08709 | 0.070% | 0.062% |  |
|  |  |  |  |  |  |
| ***Lipid Transport (GO:0006869)*** |  |  |  |  |  |
| Apolipoprotein A-I | Apoa1 | Q00623 |  | 0.023% | [30, 31] |
| Apolipoprotein E | Apoe | P08226 | 0.007% | 0.025% | [30, 31, 50] |
| Fatty acid-binding protein, liver | Fabp1 | P12710 | 0.132% | 0.133% |  |
| Perilipin-2 | Plin2 | P43883 | 0.077% | 0.297% | [50] |
| Non-specific lipid-transfer protein | Scp2 | P32020 | 0.016% | 0.048% | [29, 32, 50] |
|  |  |  |  |  |  |
| ***Other*** |  |  |  |  |  |
| Actin, cytoplasmic 1 | Actb | P60710 | 0.072% | 0.143% | [24, 27, 30, 31, 50, 62] |
| Serum albumin | Alb | P07724 | 0.280% | 0.170% | [22, 30, 31, 50] |
| Annexin A5 | Anxa5 | P48036 |  | 0.017% | [30] |
| Liver carboxylesterase 31 | Ces3a | Q63880 | 0.060% | 0.068% |  |
| Clathrin heavy chain 1 | Cltc | Q68FD5 | 0.019% | 0.093% | [26-32] |
| D-dopachrome decarboxylase | Ddt | O35215 | 0.039% | 0.047% |  |
| Elongation factor 1-alpha 1 | Eef1a1 | P10126 | 0.019% | 0.054% | [24, 31, 50] |
| Elongation factor 2 | Eef2 | P58252 | 0.049% | 0.045% |  |
| Ferritin light chain 1 | Ftl1 | P29391 | 0.047% | 0.046% |  |
| Histone H2A type 1 | Hist1h2ab | P22752 | 0.024% | 0.018% | [50] |
| Histone H2B type 1-F/J/L | Hist1h2bf | P10853 | 0.016% | 0.009% |  |
| Ribonuclease UK114 | Hrsp12 | P52760 | 0.054% | 0.048% |  |
| Interferon-inducible GTPase 1 | Iigp1 | Q9QZ85 |  | 0.006% |  |
| Methyltransferase-like protein 7B | Mettl7b | Q9DD20 | 0.053% | 0.076% | [27, 28, 50] |
| Myosin-9 | Myh9 | Q8VDD5 | 0.010% | 0.044% |  |
| Myosin light  polypeptide 6 | Myl6 | Q60605 |  | 0.009% | [50] |
| Protein NDRG2 | Ndrg2 | Q9QYG0 | 0.007% | 0.016% | [24, 50] |
| Parathymosin | Ptms | Q9D0J8 | 0.009% |  |  |
| Ras-related  protein Rab-14 | Rab14 | Q91V41 |  | 0.008% | [23, 26, 27, 32, 50] |
| Regucalcin | Rgn | Q64374 | 0.107% | 0.131% |  |
| SEC14-like protein 2 | Sec142 | Q99J08 | 0.027% | 0.027% |  |
| Selenide,  water dikinase 2 | Sephs2 | P97364 | 0.004% |  |  |
| Alpha-1-antitrypsin 1 | Serpina1a | P07758 | 0.020% | 0.015% |  |
| Serine protease inhibitor A3K | Serpina3k | P07759 |  | 0.007% |  |
| Tetratricopeptide repeat protein | Ttc36 | Q8VBW8 | 0.003% |  |  |
| Tubulin alpha-1C chain | Tuba1c | P68373 | 0.063% | 0.060% | [24, 30, 50] |
| Tubulin beta-4B chain | Tubb4b | P68372 | 0.074% | 0.062% |  |
|  |  |  |  |  |  |
| ***Redox/Detox (GO:0055114/ GO:0006805)*** |  |  |  |  |  |
| Alcohol  dehydrogenase 1 | Adh1 | P00329 | 0.123% | 0.082% |  |
| Alcohol dehydrogenase [NADP+] | Akr1a1 | Q9JII6 | 0.018% | 0.009% |  |
| Retinal dehydrogenase 1 | Aldh1a1 | P24549 | 0.110% | 0.098% |  |
| Cytosolic 10-formyltetrahydrofolate dehydrogenase | Aldh1l1 | Q8R0Y6 | 0.200% | 0.197% |  |
| Aldehyde dehydrogenase, mitochondrial | Aldh2 | P47738 |  | 0.013% | [24, 50] |
| Aldehyde dehydrogenase family 8 member A1 | Aldh8a1 | Q8BH00 | 0.055% | 0.050% |  |
| Catalase | Cat | P24270 | 0.022% | 0.031% | [24, 30] |
| Cytochrome P450 2D10 | Cyp2d10 | P24456 | 0.041% | 0.036% |  |
| Cytochrome P450 2D9 | Cyp2d9 | P11714 | 0.003% |  |  |
| Cytochrome P450 2F2 | Cyp2f2 | P33267 |  | 0.008% |  |
| Cytochrome P450 4A14 | Cyp4a14 | O35728 |  | 0.014% |  |
| Dehydrogenase/  reductase SDR family member 1 | Dhrs1 | Q99L04 | 0.046% | 0.120% | [23-26, 50, 62] |
| Glyceraldehyde-3-phosphate dehydrogenase | Gapdh | P16858 | 0.110% | 0.079% | [62] |
| Glyoxylate reductase/ hydroxypyruvate reductase | Grhpr | Q91Z53 |  | 0.010% |  |
| L-gulonolactone oxidase | Gulo | P58710 |  | 0.024% | [28] |
| 17-beta-hydroxysteroid dehydrogenase 13 | Hsd17b13 | Q8VCR2 | 0.025% | 0.137% | [30] |
| 3 beta-hydroxysteroid dehydrogenase | Hsd3b3 | P26150 |  | 0.025% | [62] |
| NADP-dependent malic enzyme | Me1 | P06801 | 0.035% | 0.007% |  |
| Pterin-4-alpha-carbinolamine dehydratase | Pcbd1 | P61458 | 0.026% | 0.017% |  |
| Peroxiredoxin-1 | Prdx1 | P35700 | 0.143% | 0.140% | [24] |
| Peroxiredoxin-5, mitochondrial | Prdx5 | P99029 | 0.009% | 0.007% | [62] |
| Superoxide dismutase [Cu-Zn] | Sod1 | P08228 | 0.067% | 0.008% |  |
| UDP-glucuronosyltransferase 1-1 | Ugt1a1 | Q63886 |  | 0.006% |  |
|  |  |  |  |  |  |
| ***Transport (GO:0006810)*** |  |  |  |  |  |
| Major urinary protein 20 | Mup20 | Q5FW60 | 0.007% | 0.011% |  |
| Major urinary protein 6 | Mup6 | P02762 | 0.022% | 0.013% |  |
| Selenium-binding  protein 2 | Selenbp2 | Q63836 | 0.109% | 0.144% |  |
| Serotransferrin | Tf | Q921I1 | 0.003% | 0.014% | [22] |
| Transitional endoplasmic reticulum ATPase | Vcp | Q01853 | 0.016% | 0.024% | [22, 24, 32] |
|  |  |  |  |  |  |
| ***Nucleotide Metabolism (GO:0006975)*** |  |  |  |  |  |
| Putative L-aspartate dehydrogenase | Aspdh | Q9DCQ2 | 0.004% | 0.008% |  |
| 3-hydroxyanthranilate 3,4-dioxygenase | Haao | Q78JT3 | 0.052% | 0.017% |  |
| Nicotinate phosphoribosyl-transferase | Naprt1 | Q8CC86 | 0.003% |  |  |
