# Supplementary material for: Dynamic Regulation of Hepatic Lipid Droplet Properties by Diet
Source: PLoS One. 2013 Jul 11;8(7):e67631. doi: 10.1371/journal.pone.0067631 (PMC3708958; doi:10.1371/journal.pone.0067631)
Supplement: Table S3 — Common CLD Associated Proteins. (DOCX) [file pone.0067631.s003.docx]

| **Table S3. Common CLD Associated Proteins** | | |
| --- | --- | --- |
| **Protein Name** | **Gene** | **Uniprot ID** |
| ***Amino Acid Metabolism (GO:0006520)*** |  |  |
| Carbonic anhydrase 3 | Ca3 | P16015 |
| Carbamoyl-phosphate synthase [ammonia], mitochondrial | Cps1 | Q8C196 |
|  |  |  |
| ***Protein Metabolism (GO:0044267)* (Chaperones)** |  |  |
| Endoplasmin | Hsp90b1 | P08113 |
| Heat shock protein HSP 90-beta | Hsp90ab1 | P11499 |
| 78 kDa glucose-regulated protein | Hspa5 | P20029 |
| Heat shock cognate 71 kDa protein | Hspa8 | P63017 |
| Protein disulfide-isomerase | P4hb | P09103 |
| Protein disulfide-isomerase A3 | Pdia3 | P27773 |
| Protein disulfide-isomerase A6 | Pdia6 | Q922R8 |
| Peptidyl-prolyl cis-trans isomerase A | Ppia | P17742 |
|  |  |  |
| ***Carbohydrate Meatbolism (GO:0005975)*** |  |  |
| Fructose-bisphosphate aldolase B | Aldob | Q91Y97 |
| Alpha-enolase | Eno1 | P17182 |
| Malate dehydrogenase, cytoplasmic | Mdh1 | P14152 |
| Phosphoglycerate kinase 1 | Pgk1 | P09411 |
| Triosephosphate isomerase | Tpi1 | P17751 |
|  |  |  |
| ***Glutathione Metabolism (GO:0006749)*** |  |  |
| Glutathione S-transferase P 1 | Gstp1 | P19157 |
|  |  |  |
| ***Lipid Metabolism (GO:0006629)*** |  |  |
| CGI58 | Abhd5 | Q9DBL9 |
| 3-ketoacyl-CoA thiolase A, peroxisomal | Acaa1a | Q921H8 |
| Acetyl-CoA acetyltransferase, cytosolic | Acat2 | Q8CAY6 |
| Long-chain-fatty-acid--CoA ligase 1 | Acsl1 | P41216 |
| Estradiol 17 beta-dehydrogenase 5 | Akr1c6 | P70694 |
| ATP synthase subunit alpha, mitochondrial | Atp5a1 | Q03265 |
| ATP synthase subunit beta, mitochondrial | Atp5b | P56480 |
| Carboxylesterase 3 | Ces1d | Q8VCT4 |
| Cytochrome b5 | Cyb5a | P56395 |
| NADH-cytochrome b5 reductase 3 | Cyb5r3 | Q9DCN2 |
| Fatty acid synthase | Fasn | P19096 |
| Monoglyceride lipase | Mgll | O35678 |
|  |  |  |
| ***Lipid Transport (GO:0006869)*** |  |  |
| Apolipoprotein E | Apoe | P08226 |
| Apolipoprotein A-I | Apoa1 | Q00623 |
| Perilipin-2 | Plin2 | P43883 |
| Non-specific lipid-transfer protein | Scp2 | P32020 |
|  |  |  |
| ***Other*** |  |  |
| Actin, cytoplasmic 1 | Actb | P60710 |
| Serum albumin | Alb | P07724 |
| Annexin A5 | Anxa5 | P48036 |
| Clathrin heavy chain 1 | Cltc | Q68FD5 |
| Elongation factor 1-alpha 1 | Eef1a1 | P10126 |
| Histone H2A type 1 | Hist1h2ab | P22752 |
| Methyltransferase-like protein 7B | Mettl7b | Q9DD20 |
| Myosin light polypeptide 6 | Myl6 | Q60605 |
| Protein NDRG2 | Ndrg2 | Q9QYG0 |
| Ras-related protein Rab-14 | Rab14 | Q91V41 |
| Tubulin alpha-1C chain | Tuba1c | P68373 |
|  |  |  |
| ***Redox/Detox (GO:0055114/ GO:0006805)*** |  |  |
| Aldehyde dehydrogenase, mitochondrial | Aldh2 | P47738 |
| Catalase | Cat | P24270 |
| Dehydrogenase/reductase SDR family member 1 | Dhrs1 | Q99L04 |
| Glyceraldehyde-3-phosphate dehydrogenase | Gapdh | P16858 |
| L-gulonolactone oxidase | Gulo | P58710 |
| 17-beta-hydroxysteroid dehydrogenase 13 | Hsd17b13 | Q8VCR2 |
| 3 beta-hydroxysteroid dehydrogenase | Hsd3b3 | P26150 |
| Peroxiredoxin-1 | Prdx1 | P35700 |
| Peroxiredoxin-5, mitochondrial | Prdx5 | P99029 |
|  |  |  |
| ***Transport (GO:0006810)*** |  |  |
| Serotransferrin | Tf | Q921I1 |
| Transitional endoplasmic reticulum ATPase | Vcp | Q01853 |
