# Supplementary material for: Dynamic Regulation of Hepatic Lipid Droplet Properties by Diet
Source: PLoS One. 2013 Jul 11;8(7):e67631. doi: 10.1371/journal.pone.0067631 (PMC3708958; doi:10.1371/journal.pone.0067631)
Supplement: Table S4 — Liver Specific CLD Associated Proteins. (DOCX) [file pone.0067631.s004.docx]

| **Table S4. Liver Specific CLD Associated Proteins** | | |
| --- | --- | --- |
| **Protein Name** | **Gene** | **Uniprot ID** |
| ***Amino Acid Metabolism (GO:0006520)*** |  |  |
| Adenosylhomocysteinase | Ahcy | P50247 |
| 4-trimethylaminobutyraldehyde dehydrogenase | Aldh9a1 | Q9JLJ2 |
| Arginase-1 | Arg1 | Q61176 |
| Argininosuccinate lyase | Asl | Q91YI0 |
| Argininosuccinate synthase | Ass1 | P16460 |
| Betaine--homocysteine S-methyltransferase 1 | Bhmt | O35490 |
| Cystathionine gamma-lyase | Cth | Q8VCN5 |
| Dihydropyrimidinase | Dpys | Q9EQF5 |
| Fumarylacetoacetase | Fah | P35505 |
| Formimidoyltransferase-cyclodeaminase | Ftcd | Q91XD4 |
| Glutamine synthetase | Glul | P15105 |
| Glycine N-methyltransferase | Gnmt | Q9QXF8 |
| Aspartate aminotransferase, cytoplasmic | Got1 | P05201 |
| Aspartate aminotransferase, mitochondrial | Got2 | P05202 |
| Alanine aminotransferase 1 | Gpt | Q8QZR5 |
| Maleylacetoacetate isomerase | Gstz1 | Q9WVL0 |
| Histidine ammonia-lyase | Hal | P35492 |
| 4-hydroxyphenylpyruvate dioxygenase | Hpd | P49429 |
| Cytosol aminopeptidase | Lap3 | Q9CPY7 |
| S-adenosylmethionine synthase isoform type-1 | Mat1a | Q91X83 |
| C-1-tetrahydrofolate synthase, cytoplasmic | Mthfd1 | Q922D8 |
| Phenylalanine-4-hydroxylase | Pah | P16331 |
| L-serine dehydratase/L-threonine deaminase | Sds | Q8VBT2 |
| Sepiapterin reductase | Spr | Q64105 |
| Urocanate hydratase | Uroc1 | Q8VC12 |
| Homogentisate 1,2-dioxygenase | Hgd | O09173 |
|  |  |  |
| ***Protein Metabolism (GO:0044267)* (Chaperones)** |  |  |
| Phenazine biosynthesis-like domain-containing protein 1 | Pbld1 | Q9DCG6 |
|  |  |  |
| ***Carbohydrate Meatbolism (GO:0005975)*** |  |  |
| Isocitrate dehydrogenase [NADP] cytoplasmic | Idh1 | O88844 |
| Cytoplasmic aconitate hydratase | Aco1 | P28271 |
| Pancreatic alpha-amylase | Amy2 | P00688 |
| Bifunctional ATP-dependent dihydroxyacetone kinase /FAD-AMP lyase (cyclizing) | Dak | Q8VC30 |
| Fructose-1,6-bisphosphatase 1 | Fbp1 | Q9QXD6 |
| 1,4-alpha-glucan-branching enzyme | Gbe1 | Q9D6Y9 |
| Ketohexokinase | Khk | P97328 |
| L-lactate dehydrogenase A chain | Ldha | P06151 |
| Phosphoglycerate mutase 1 | Pgam1 | Q9DBJ1 |
| Phosphoglucomutase-1 | Pgm1 | Q9D0F9 |
| Pyruvate kinase isozymes R/L | Pklr | P53657 |
| Glycogen phosphorylase, liver form | Pygl | Q9ET01 |
| Sorbitol dehydrogenase | Sord | Q64442 |
| Transketolase | Tkt | P40142 |
| UTP--glucose-1-phosphate uridylyltransferase | Ugp2 | Q91ZJ5 |
|  |  |  |
| ***Glutathione Metabolism (GO:0006749)*** |  |  |
| Lactoylglutathione lyase | Glo1 | Q9CPU0 |
| Glutathione peroxidase 1 | Gpx1 | P11352 |
| Glutathione S-transferase A3 | Gsta3 | P30115 |
| Glutathione S-transferase Mu 1 | Gstm1 | P10649 |
|  |  |  |
| ***Lipid Metabolism (GO:0006629)*** |  |  |
| Peroxiredoxin-6 | Prdx6 | O08709 |
| ATP-binding cassette sub-family D member 3 | Abcd3 | P55096 |
| 3-ketoacyl-CoA thiolase B, peroxisomal | Acaa1b | Q8VCH0 |
| Very long-chain specific acyl-CoA dehydrogenase | Acadvl | P50544 |
| ATP-citrate synthase | Acly | Q91V92 |
| Peroxisomal acyl-coenzyme A oxidase 1 | Acox1 | Q9R0H0 |
| Cytochrome P450 2E1 | Cyp2e1 | Q05421 |
| Peroxisomal bifunctional enzyme | Ehhadh | Q9DBM2 |
| Epoxide hydrolase 2 | Ephx2 | P34914 |
| Hydroxymethylglutaryl-CoA synthase | Hmgcs2 | P54869 |
| Estradiol 17-beta-dehydrogenase 11 | Hsd17b11 | Q9EQ06 |
| Phosphoenolpyruvate carboxykinase, cytosolic [GTP] | Pck1 | Q9Z2V4 |
| Inorganic pyrophosphatase | Ppa1 | Q9D819 |
|  |  |  |
| ***Lipid Transport (GO:0006869)*** |  |  |
| Fatty acid-binding protein, liver | Fabp1 | P12710 |
|  |  |  |
| ***Nucleotide Metabolism (GO:0006975)*** |  |  |
| Putative L-aspartate dehydrogenase | Aspdh | Q9DCQ2 |
| Nicotinate phosphoribosyltransferase | Naprt1 | Q8CC86 |
| 3-hydroxyanthranilate 3,4-dioxygenase | Haao | Q78JT3 |
|  |  |  |
| ***Other*** |  |  |
| Liver carboxylesterase 31 | Ces3a | Q63880 |
| Elongation factor 2 | Eef2 | P58252 |
| Ferritin light chain 1 | Ftl1 | P29391 |
| Histone H2B type 1-F/J/L | Hist1h2bf | P10853 |
| Ribonuclease UK114 | Hrsp12 | P52760 |
| Interferon-inducible GTPase 1 | Iigp1 | Q9QZ85 |
| Myosin-9 | Myh9 | Q8VDD5 |
| Parathymosin | Ptms | Q9D0J8 |
| Regucalcin | Rgn | Q64374 |
| SEC14-like protein 2 | Sec142 | Q99J08 |
| Selenide, water dikinase 2 | Sephs2 | P97364 |
| Alpha-1-antitrypsin 1 | Serpina1a | P07758 |
| Serine protease inhibitor A3K | Serpina3k | P07759 |
| Tetratricopeptide repeat protein | Ttc36 | Q8VBW8 |
| Tubulin beta-4B chain | Tubb4b | P68372 |
| D-dopachrome decarboxylase | Ddt | O35215 |
|  |  |  |
| ***Redox/Detox (GO:0055114/ GO:0006805)*** |  |  |
| Alcohol dehydrogenase 1 | Adh1 | P00329 |
| Alcohol dehydrogenase [NADP+] | Akr1a1 | Q9JII6 |
| Retinal dehydrogenase 1 | Aldh1a1 | P24549 |
| Aldehyde dehydrogenase family 8 member A1 | Aldh8a1 | Q8BH00 |
| Cytochrome P450 2D10 | Cyp2d10 | P24456 |
| Cytochrome P450 2D9 | Cyp2d9 | P11714 |
| Cytochrome P450 2F2 | Cyp2f2 | P33267 |
| Cytochrome P450 4A14 | Cyp4a14 | O35728 |
| Glyoxylate reductase/hydroxypyruvate reductase | Grhpr | Q91Z53 |
| NADP-dependent malic enzyme | Me1 | P06801 |
| Pterin-4-alpha-carbinolamine dehydratase | Pcbd1 | P61458 |
| Superoxide dismutase [Cu-Zn] | Sod1 | P08228 |
| UDP-glucuronosyltransferase 1-1 | Ugt1a1 | Q63886 |
| Cytosolic 10-formyltetrahydrofolate dehydrogenase | Aldh1l1 | Q8R0Y6 |
|  |  |  |
| ***Transport (GO:0006810)*** |  |  |
| Major urinary protein 6 | Mup6 | P02762 |
| Major urinary protein 20 | Mup20 | Q5FW60 |
| Selenium-binding protein 2 | Selenbp2 | Q63836 |
